# Co-immunoprecipitation with Tau Isoform-specific Antibodies Reveals Distinct Protein Interactions and Highlights a Putative Role for 2N Tau in Disease*[Fn FN2]

**DOI:** 10.1074/jbc.M115.641902

**Published:** 2016-02-09

**Authors:** Chang Liu, Xiaomin Song, Rebecca Nisbet, Jürgen Götz

**Affiliations:** From the ‡ Sydney Medical School, Brain and Mind Research Institute, University of Sydney, Camperdown, New South Wales 2050,; the § Australian Proteome Analysis Facility, Macquarie University (Sydney), New South Wales 2109, and; the ¶ Clem Jones Centre for Ageing Dementia Research, Queensland Brain Institute, University of Queensland, St. Lucia Campus (Brisbane), Queensland 4072, Australia

**Keywords:** Alzheimer disease, apolipoprotein, protein/protein interaction, Tau protein (Tau), Tauopathy

## Abstract

Alternative splicing generates multiple isoforms of the microtubule-associated protein Tau, but little is known about their specific function. In the adult mouse brain, three Tau isoforms are expressed that contain either 0, 1, or 2 N-terminal inserts (0N, 1N, and 2N). We generated Tau isoform-specific antibodies and performed co-immunoprecipitations followed by tandem mass tag multiplexed quantitative mass spectrometry. We identified novel Tau-interacting proteins of which one-half comprised membrane-bound proteins, localized to the plasma membrane, mitochondria, and other organelles. Tau was also found to interact with proteins involved in presynaptic signal transduction. MetaCore analysis revealed one major Tau interaction cluster that contained 33 Tau pulldown proteins. To explore the pathways in which these proteins are involved, we conducted an ingenuity pathway analysis that revealed two significant overlapping pathways, “cell-to-cell signaling and interaction” and “neurological disease.” The functional enrichment tool DAVID showed that in particular the 2N Tau-interacting proteins were specifically associated with neurological disease. Finally, for a subset of Tau interactions (apolipoprotein A1 (apoA1), apoE, mitochondrial creatine kinase U-type, β-synuclein, synaptogyrin-3, synaptophysin, syntaxin 1B, synaptotagmin, and synapsin 1), we performed reverse co-immunoprecipitations, confirming the preferential interaction of specific isoforms. For example, apoA1 displayed a 5-fold preference for the interaction with 2N, whereas β-synuclein showed preference for 0N. Remarkably, a reverse immunoprecipitation with apoA1 detected only the 2N isoform. This highlights distinct protein interactions of the different Tau isoforms, suggesting that they execute different functions in brain tissue.

## Introduction

Tau belongs to the family of microtubule-associated proteins (MAPs)^2^ that act in concert with heterodimers of α- and β-tubulin to assemble microtubules (1). The members of the MAP family were named according to the three major size classes of polypeptides: MAP1 (>250 kDa), MAP2 (∼200 kDa), and Tau (50–70 kDa) (1, 2). MAP2 and Tau are expressed together in most neurons, where they partially segregate into separate subcellular compartments, when maturation is completed. MAP2 is largely found in dendrites, whereas Tau is concentrated in axons (3). Tau has also been found in astrocytes and oligodendrocytes under physiological conditions, although at relatively low levels (4).

Tau plays a crucial role in neuro-degeneration and as such has become a target of therapeutic interventions (5). In Alzheimer disease (AD) and other diseases collectively termed tauopathies, Tau becomes hyperphosphorylated and forms insoluble aggregates. In this process, Tau exerts its toxicity by various integrated mechanisms as revealed in transgenic animal models that model aspects of the human disease (6, 7). When Tau is routinely studied, it is often treated as if it were a single protein, whereas in fact it exists as multiple isoforms and phospho-species (8). Not only does it contain 85 sites that can be potentially phosphorylated (five tyrosine and 80 serine/threonine residues) (9), but it also exists as several isoforms whose functions are incompletely understood. The central nervous system of adult mice expresses three low molecular weight Tau isoforms that are generated by alternative splicing and have either 0, 1, or 2 N-terminal inserts (0N, 1N, and 2N), as well as four (4R) microtubule-binding domains. In contrast, in the developing embryonic brain, the 0N3R isoform predominates (10, 11). Together, this implies that the different Tau species must interact with specific subsets of proteins and execute specific cellular functions.

In addition to interacting with cytoskeletal proteins such as tubulin that forms the microtubules (12–16), Tau has previously been shown to interact with other protein classes. They include kinases and phosphatases such as protein phosphatase 2A (PP2A) (17), extracellular proteins such as apolipoprotein E (apoE), and membrane proteins such as the Src kinase Fyn (18). The latter has been shown to differ in its interaction depending on the presence of three or four microtubule-binding repeats (3R or 4R) and whether or not Tau carries pathogenic mutations found in familial forms of frontotemporal dementia (19). We have previously demonstrated in wild-type mice that the 0N, 1N, and 2N isoforms of Tau show a distinct subcellular distribution suggesting subunit-specific functions (11). The finding that 1N Tau is enriched in the nucleus suggested to us that a deregulation of Tau's presumable nuclear functions could potentially contribute to pathological conditions. Tau is ideally positioned to regulate cellular functions because there is accumulating evidence that it functions as a major scaffolding protein (20), being able to bind at least two signaling proteins, and thereby localize signaling molecules and transduction pathways to defined subcellular locations (21).

To gain a deeper understanding of the Tau isoforms differing in their N-terminal domain, we sought to use co-immunoprecipitation (co-IP) to reveal distinct interactions, using 0N, 1N, and 2N Tau-specific antibodies. We determined the identity of the interacting proteins using tandem mass tag (TMT) multiplexed quantitative mass spectrometry (MS), including Tau knock-out (KO) brain tissue and pan-Tau-specific antibodies as controls. We identified novel Tau-interacting proteins that were subjected to a bioinformatics analysis to reveal two major pathway clusters as presented below: a neurological process-related cluster, and an energy metabolism-related cluster. A specific role in neurological disease was identified for the 2N isoform of Tau. A subset of proteins was validated by reverse co-IPs and ELISA, underscoring the validity of the MS analysis.

## Experimental Procedures

### 

#### 

##### Ethics Statement and Mouse Strains

Animal experimentation was approved by the Animal Ethics Committees of the University of Queensland (approval number QBI/027/12/NHMRC). For the co-IP studies, C57BL/6J wild-type and Tau KO mice (22) were used.

##### Antibodies Used for Co-immunoprecipitation

Antibodies specific for total (M), 0N, 1N, and 2N murine Tau were used (11). The 0N antibody had been raised against the junction between exons 1 and 4 using peptide DMDHGLKAEEAGIG, the 1N antibody against the junction between exons 2 and 4 using peptide DAKSTPTAEAEEAG, the 2N antibody against the junction between exons 3 and 4 using peptide TEIPEGITAEEAGI, and the M antibody against peptide RVASKDRTGNDEKK encoded by exon 5. These antibodies have previously been shown to be specific using both Tau KO extracts and the pre-absorption with the above peptides as control (11).

##### Preparation of Recombinant Proteins

pRc/CMV plasmids containing cDNAs encoding the three murine Tau isoforms were kindly provided by Dr. Gloria Lee. They are referred to as Mtau10 (0N4R), Mtau210 (1N4R), and Mtau2310 (2N4R), respectively. The forward primer 5′-ATGGCTGACCCTCGCCAG-3′ and the reverse primer 5′-TCACAAACCCTGCTTGGCCAA-3′ were used to clone the murine Tau cDNAs into the pGEX4t1 plasmid (GE Healthcare) in-frame with an N-terminal GST tag. The murine β-synuclein and synaptophysin cDNAs in pCMV6-Kan/Neo (OriGene) were PCR-amplified using the following forward and reverse primers 5′-CACCATGGACGTGTTCATGAAGG-3′ and 5′-TTACGCCTCTGGCTCGTATTC-3′ for β-synuclein and 5′-CACCATGGACGTGGTGAATCAGC-3′ and 5′-TTACATCTGATTGGAGAAGGAGG-3′ for synaptophysin. Genes were then cloned into the pET-DEST42 vector (Life Technologies, Inc.) in-frame with a C-terminal His_6_ and V5 tag. Plasmids were transformed into One Shot® BL21^TM^ bacterial cells (Life Technologies, Inc.), and recombinant protein expression was induced with 1 mm isopropyl 1-thio-β-d-galactopyranoside for 2 h at 37 °C. The bacterial suspension was pelleted at 4,000 × *g* for 15 min at 4 °C and then resuspended in GST buffer (150 mm NaCl, 10 mm Na_2_HPO_4_, 5 mm EDTA, pH 7.4) for Tau proteins or IMAC buffer (300 mm KCl, 50 mm KH_2_PO_4_, 5 mm imidazole, pH 8.0) for β-synuclein and synaptophysin containing 0.1% Complete protease inhibitor and 0.1 mg/ml lysozyme (Sigma) followed by incubation on ice for 20 min. The cells were then subjected to repeated freeze/thawing after which they were sonicated at a 60% amplitude for 1 min (Qsonica Sonicator, Newtown, CT). The lysate was centrifuged at 16,000 × *g* for 20 min at 4 °C and filtered through a 0.22-μm syringe filter (Millipore, Billerica, MA). For the purification of recombinant Tau, the supernatant was passed over a 1-ml Bio-Scale Mini Profinity GST cartridge (Bio-Rad) equilibrated in GST buffer using a Profinia Protein Purification System (Bio-Rad) and then eluted in 20 mm glutathione, 100 mm Tris, 10 mm EDTA, pH 8.0. For recombinant β-synuclein and synaptophysin purification, the filtered lysates were passed over a 1-ml Bio-Scale Mini IMAC cartridge (Bio-Rad) equilibrated in IMAC buffer and eluted in 300 mm KCl, 50 mm KH_2_PO_4_, 250 mm imidazole, pH 8.0. Eluted proteins underwent buffer exchange into 137 mm NaCl, 2.7 mm KCl, 4.3 mm Na_2_HPO_4_, 88 mm KH_2_PO_4_, pH 7.4, using a Bio-Scale Mini Bio-Gel P-6 Desalting Cartridge (Bio-Rad) and were then subjected to size exclusion chromatography using an S200 10/30 GL column (GE Healthcare) equilibrated in 1× PBS. Fractions corresponding to the protein of interest were combined and concentrated using an Amicon Ultra Filter with a 3,000-kDa molecular mass cutoff (Merck Millipore).

##### Co-immunoprecipitation and Western Blot Analysis

Whole brains were dissected from mice and stored at −80 °C. The frozen brains were washed with 5 ml of ice-cold IP washing buffer (modified Dulbecco's phosphate-buffered saline (MDPBS): 8.1 mm Na_2_HPO_4_, 1.47 mm KH_2_PO_4_, 2.7 mm KCl, and 140 mm NaCl) (23), after which they were homogenized in 10 μl/μg ice-cold IP buffer, containing 25 mm Tris-HCl, pH 7.4, 150 mm NaCl, 1 mm EDTA, 1% Nonidet P-40, 5% glycerol, protease inhibitors (Complete Mini, Roche Applied Science, Basel, Switzerland), and phosphatase inhibitors (PhoSTOP, Roche Applied Science), with a micro-pestle 20 times and passed through an insulin syringe 10 times. The lysate was then incubated on ice for 10 min with shaking to solubilize the proteins. The homogenate was centrifuged for 40 min at 20,000 × *g* at 4 °C. The supernatant was transferred to a new tube, and the pellet containing cell debris was discarded. The transferred supernatant was centrifuged again for 10 min at 20,000 × *g* at 4 °C, after which the supernatant was transferred to a new tube.

For the pre-cleaning of the lysate, protein G-Sepharose 4 Fast Flow beads (GE Healthcare) were first washed three times in ice-cold MDPBS, pH 7.4. The brain supernatants were then mixed with the pre-washed beads and incubated on a rocking platform overnight at 4 °C, after which the mixture was centrifuged at 600 × *g* for 5 min, and the supernatant was transferred to a new tube that was labeled “input.”

To prepare the antibody-bound beads, an appropriate amount (∼20 μg) of non-immune sera and antibodies (mouse, 0N, 1N, 2N, and Dako (Agilent Technologies, Glostrup, Denmark)) were incubated together with 120 μl of washed beads in IP binding buffer (MDPBS, pH 5.5) and incubated on a rocking platform overnight at 4 °C (Fig. 2). The mixture was then centrifuged at 600 × *g* for 5 min, followed by three washes of the antibody-bound beads in ice-cold IP buffer.

To immunoprecipitate the protein complexes, 1 mg of the pre-cleaned supernatants was added to the antibody-bound beads and incubated on a rocking platform overnight at 4 °C. After centrifugation for 5 min at 600 × *g* at 4 °C, the supernatant was carefully removed and saved as “flow-through” (FT). The beads were washed extensively (by turning the tube upside down 10 times) with IP washing buffer to obtain complexes of proteins bound to the antibody-coated beads (saved as IP). The set of samples destined for Western blot analysis was directly boiled in 1× SDS-PAGE sample buffer (50 mm Tris-HCl, pH 6.8, 2% SDS, 10% glycerol, 1% β-mercaptoethanol, 12.5 mm EDTA, and 0.02% bromphenol blue) (24), followed by SDS-PAGE and Western blot analysis. The set of samples destined for MS analysis was kept at −80 °C until further use.

##### TMT Multiplexed Quantitative Mass Spectrometry

For a flow-chart of the mass spectrometric analysis, see Fig. 2. The TMT analysis was done with three sets of biological replicates. The IP samples and the corresponding TMT labels are listed in Table 1. For the tryptic digestion, 100 μl of tetraethylammonium bromide (Sigma) was added to each IP sample. The samples were reduced with 10 mm tris(2-carboxyethyl)phosphine (Thermo Scientific, Rockford, IL) at room temperature for 15 min and alkylated with 20 mm iodoacetamide at room temperature for 30 min in the dark. The samples then underwent a 16-h digestion with 6 μg of trypsin (Sigma, catalog no. T6567) at 37 °C.

The peptides were retrieved and separated from the beads by centrifugation at 14,000 × *g* for 20 min using spin filters (Millipore, Billerica, MA). The concentration was determined using Direct Detect (Millipore). The protein concentration was estimated, and 50 μg of each digested sample was labeled with the TMT kit (Thermo Scientific, catalog no. 90113). Briefly, the peptides were incubated with TMT reagents for 1 h at room temperature following the manufacturer's instructions, after which 8 μl of 50% hydroxylamine was added to quench the reaction.

Following TMT labeling, 2 μl from each sample was pooled and cleaned using a detergent removal spin column (Thermo Scientific, catalog no. 87777), dried down, and resuspended in 20 μl of 0.1% formic acid (ProteoChem, Loves Park, IL, catalog no. LC6201). This sample was subjected to nano-LC-ESI MS/MS analysis. The labeled and cleaned peptides were separated using an Easy nano-LC 1000 system (Thermo Scientific) that was directly interfaced with a Thermo Q Exactive benchtop mass spectrometer. An “in-house” packed solid core Halo C18 resin column (160 Å, 2.7 μm, 75 μm × 3.5 cm) was used as a peptide trap column. The analytical column was an in-house packed solid core Halo C18 resin column (160 Å, 2.7 μm, 75 μm × 10 cm). The following buffers were used: mobile phase buffer A consisted of 0.1% formic acid, and mobile phase buffer B consisted of 99.9% acetonitrile and 0.1% formic acid. A 10-μl sample was injected into the peptide trap column that had been pre-equilibrated with buffer A and desalted with 20 μl of 0.1% formic acid. After desalting, the peptide trap column was switched on line to the analytical column. The peptides were eluted from the column using a linear solvent gradient, with steps from 1 to 30% buffer B for 170 min and 30–85% of buffer B for 2 min, followed by holding at 85% of buffer B for 8 min with a flow rate of 300 nl/min across the gradient.

The column eluent was directed into a nanospray ionization source of the mass spectrometer. A 1.6-kV electrospray voltage was applied via a liquid junction upstream of the column. The mass spectrum was scanned across a mass range of 350–2000 *m*/*z* at a resolution of 70,000. Automated peak recognition, 10-s dynamic exclusion, and tandem MS of the top 10 most intense precursor ions at 30% normalized collision energy were performed using Xcalibur software (Thermo Scientific).

The MS/MS spectra from each LC-MS/MS run were searched with Proteome Discoverer version 1.3 using Mascot version 2.4 (Matrix Science, London, UK) against the house mouse protein database NCBInr (National Center for Biotechnology Information, Bethesda, sequence entry 173591 for *Mus musculus*). The search criteria were as follows: full tryptic specificity was required; one missed cleavage was allowed; oxidation (M), carbamidomethylation (C), and TMT 6plex (K) were set as variable modifications; TMT labeling (N-terminal) was set as a fixed modification; precursor ion mass tolerances were set at 10 ppm; and the fragment ion mass tolerance at 0.1 Da for all MS2 spectra was acquired. Relative protein quantification was also performed using the Proteome Discoverer software based on peptide TMT tag ion intensities. The MS/MS analysis revealed (as was expected from the variable protein concentration readings) that for the pooled 2-μl samples, there was a significant difference for each TMT's “channel” reflecting the total amount of labeled protein in the respective reactions. Therefore, new samples were pooled from the eight labeled samples adjusting volumes accordingly, using total protein quantity ratios determined by Proteome Discovery quantification software as a guide. The nano-LC ESI MS/MS data were acquired for the new pooled samples. These data were processed with Proteome Discovery, and the relative total protein quantity ratios between channels were confirmed to be within a 20% difference, which was then corrected to obtain the same quantity (Fig. 2).

##### Bioinformatic Analysis

Pathway analysis was performed using the MetaCore and IPA to assess the degree of interactions among the identified proteins. KEGG pathways enrichment, GO (Gene Ontology) cellular components enrichment, and GO biological process enrichment were done using DAVID (25). The Fisher's exact test was used for analysis of the significance of enrichment. The following provides additional information about these analysis tools. (i) Metacore (Thomson Reuters) is an integrated software suite for the functional analysis of proteomic data based on a high quality, manually curated database. (ii) IPA (Qiagen Redwood City) is used to analyze, integrate, and understand proteomic data with a focus on capturing relevant networks, (iii) DAVID (david.ncifcrf.gov) is an acronym for Database for Annotation, Visualization, and Integrated Discovery and is an open-source, web-accessible program to provide a comprehensive set of functional annotations. (iv) KEGG (Kyoto Encyclopedia of Genes and Genomes) is a database resource with molecular level information, particularly large scale molecular datasets generated by genome sequencing and other high throughput experimental technologies. (v) GO provides a controlled vocabulary of terms for describing gene product characteristics and gene product data. Pathway analysis was done by multiple comparisons algorithms using Fisher's exact test. *p* values were adjusted for multiple testing errors with the Benjamini-Hochberg critical value for a false discovery rate of 0.1.

##### Reverse Co-immunoprecipitations

For reverse co-IPs, protein G-Sepharose 4 Fast Flow beads (GE Healthcare) were pre-bound with 4 μg of the following antibodies, respectively: anti-mitochondrial creatine kinase U-type (MtCK, Abcam, San Francisco, catalog no. ab76506); anti-β-synuclein (Abcam, catalog no. ab76111); anti-apoE (Abcam, catalog no. ab1906); anti-apoAI (Santa Cruz Biotechnology, Dallas, TX, catalog no. sc-30089); anti-syntaxin 1B (Synaptic Systems, Göttingen, Germany, catalog no. 110402); anti-synapsin 1 (Abcam, catalog no. ab8); anti-synaptogyrin-3 (Santa Cruz Biotechnology, catalog no. sc-68936); anti-synaptophysin (Santa Cruz Biotechnology, catalog no. sc-9116); and anti-synaptotagmin (Millipore, catalog no. MAB5200). These samples were then incubated with 1 mg of lysate at 4 °C overnight. To verify the binding preference of Tau isoforms, dephosphorylated lysates were used adapting a dephosphorylation protocol obtained from New England Biolabs. Beads were washed extensively with IP washing buffer and eluted in 2× SDS-PAGE sample buffer for subsequent Western blot analysis. Blots were probed with the following antibodies: anti-creatine kinase U-type; mitochondrial (MtCK); anti-β-synuclein; anti-apoE; anti-apoAI; anti-syntaxin 1B; anti-synapsin 1; anti-synaptogyrin-3; anti-synaptophysin; anti-synaptotagmin, and anti-Tau. For quantification, ImageJ was used.

##### Recombinant Protein ELISA

Direct binding of recombinant Tau isoforms was determined using ELISA. All wash steps consisted of three 1-min PBS washes. Immuno-96-MicroWell plates (Nunc) were coated overnight at 4 °C with recombinant 0N, 1N, and 2N Tau at 10 μg/ml in PBS. Plates were washed and then blocked with 3% BSA in PBS for 2 h at room temperature. Recombinant β-synuclein and synaptophysin were incubated at 10 μg/ml in PBS for 1 h at room temperature, washed, and then detected with anti-V5 antibody (1:2,000; Sigma) followed by anti-mouse horseradish peroxidase antibody conjugate (1:5,000) each for 1 h at room temperature with a wash in-between. Substrate solution, One-Step Ultra 3,3′,5,5′-tetramethylbenzidine (Life Technologies, Inc.), was added to each well and incubated for 15 min. The absorbance was measured with a 450-nm filter (CLARIOstar, BMG Labtech). Measurements were performed in triplicate and analyzed after subtraction of the absorbance of wells coated with BSA.

##### Statistics for Biochemical Studies

Statistical analysis was conducted using Prism 6 software using Student's *t* test. All values are given as means ± S.E.

## Results

### 

#### 

##### Parallel Immunoprecipitation of Tau Isoform-interacting Proteins

We had previously generated Tau isoform-specific antibodies for the 0N, 1N, and 2N forms of Tau and a pan-Tau-specific antibody labeled M(ouse) (11). Here, we were interested in determining the isoform-specific interactome. To isolate proteins that either directly or indirectly interact with total Tau and specific Tau isoforms, we set up eight parallel IPs, because of the fact that there are eight TMTs available for subsequent TMT multiplexed quantitative MS (one reaction was duplicated, see Table 1). TMT is an isobaric labeling method used in quantitative proteomics by tandem MS to determine the amount of proteins from different sources in a single experiment (26). For seven reactions, we used brain lysates obtained from 2-month-old C57BL/6 wild-type mice, and for the eighth reaction we used a brain lysate from an age-matched Tau KO mouse as a negative control (127C tag). Two reactions used Dako Tau, a pan-Tau-specific antibody (tags 126 as well as 127N for internal standardization). One reaction used antibodies 0N, 1N, and 2N (with tags 129N, 130N and 131, respectively). We also ran one reaction with our pan-Tau-specific antibody M(ouse) (128C tag). An IP reaction using normal mouse serum was included as a second negative control to exclude interference due to nonspecific binding with mouse immunoglobulins (tag 128N).

**TABLE 1:** **IP samples and the corresponding TMT labels**

No.	Sample name	Input	Antibody used	TMT labeling
1	IP:Dako with C57BL/6	2-Month-old C57BL/6 mouse brain lysate (wild type)	Dako Tau	126
2	IP:Dako with C57BL/6	2-Month-old C57BL/6 mouse brain lysate (wild type)	Dako Tau	127N
3	IP:Dako with Tau KO	2-Month-old Tau knock-out mice brain lysate (Tau KO)	Dako Tau	127C
4	IP:normal serum	2-Month-old C57BL/6 mouse brain lysate (wild type)	Normal mouse serum	128N
5	IP:mouse	2-Month-old C57BL/6 mouse brain lysate (wild type)	M(ouse) Tau antibody	128C
6	IP:0N	2-Month-old C57BL/6 mouse brain lysate (wild type)	0N Tau antibody	129N
7	IP:1N	2-Month-old C57BL/6 mouse brain lysate (wild type)	1N Tau antibody	130N
8	IP:2N	2-Month-old C57BL/6 mouse brain lysate (wild type)	2N Tau antibody	131

**FIGURE 1.**
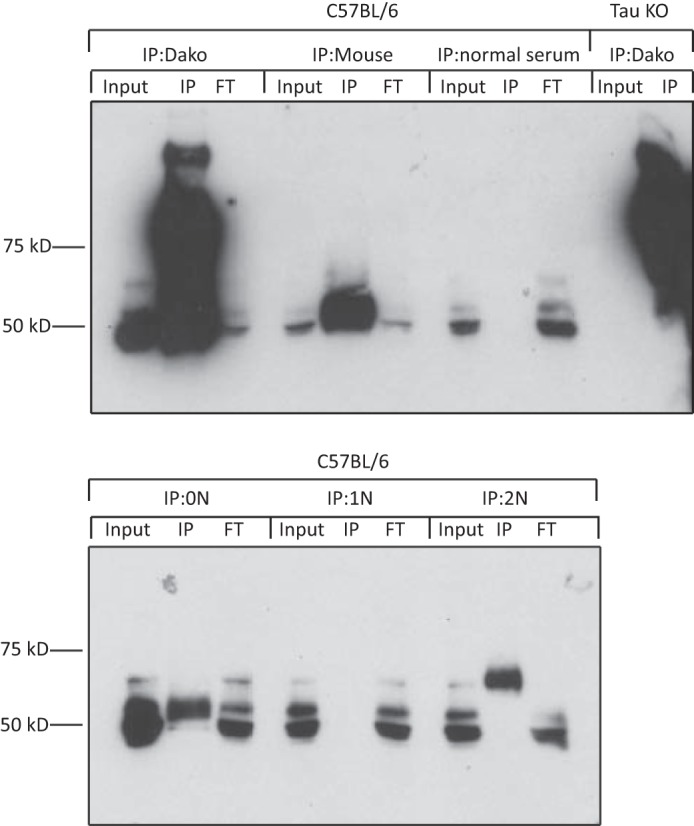
**Western blot validation of Tau immunoprecipitation reactions.** The *1st row* indicates the tissue source, and the *2nd row* indicates the antibody used for the IP (Tau isoform-specific antibodies 0N, 1N, and 2N and pan-Tau antibodies Dako Tau and M). The input, IP, and flow-through (*FT*) were loaded for each reaction. An IP from wild-type mouse brain using normal serum (*IP:normal serum*) and from a Tau KO brain using the Dao antibody (*IP:Dako with Tau KO mice*) were included as negative controls.

To validate the IP reactions, we performed Western blot analysis, demonstrating that total Tau was successfully pulled down with both Dako Tau and M. 0N and 2N Tau were also successfully and specifically pulled down with the respective isoform-specific antibodies. However, we failed to detect 1N Tau in the immunoprecipitated material by Western blotting, possibly due to the very low amount of 1N present, combined with the lower affinity of this antibody compared with 0N or 2N (Fig. 1). We did, however, detect 1N Tau by MS in this IP reaction (see below). The background on the blot is because the secondary goat anti-rabbit HRP-labeled antibody not only recognizes the rabbit anti-Tau antibody probing the blot but also the rabbit anti-Tau antibody used for the IP.

##### Identification of Tau-interacting Proteins by Mass Spectrometry

**FIGURE 2.**
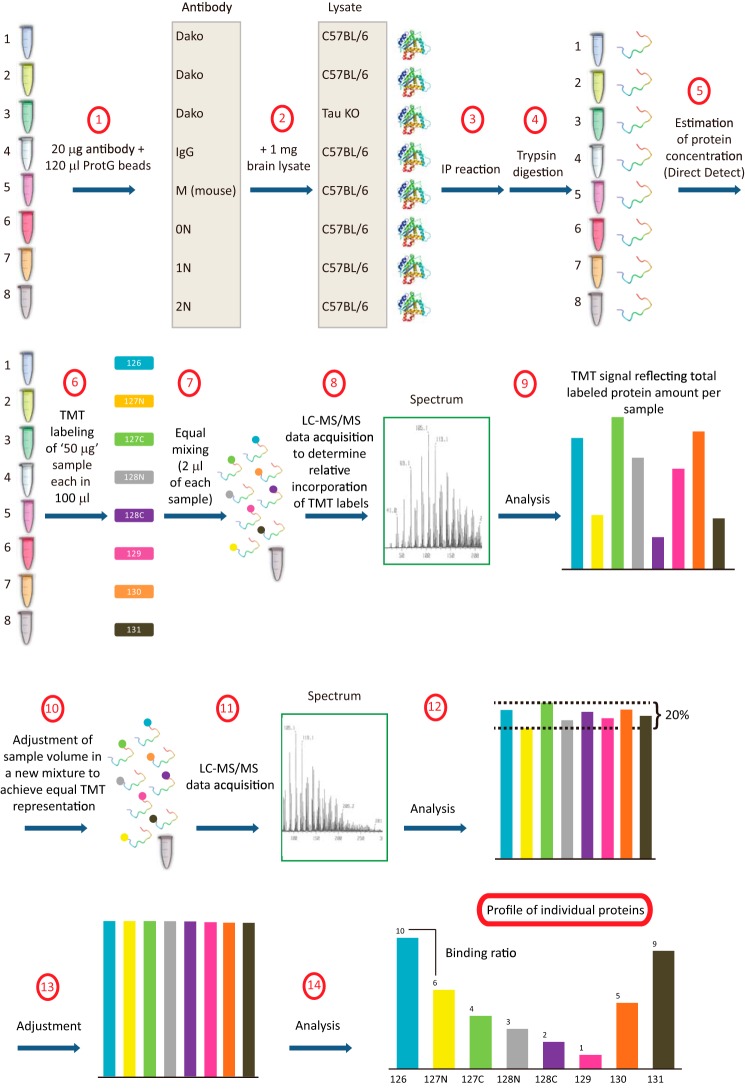
**Schematic diagram of the quantitative proteomics workflow by LC-MS/MS.** The procedure combines incubation of the indicated antibodies with protein G (*ProtG*) beads, followed by adding the indicated lysates, digestion into peptides, an estimation of the protein concentration, isobaric tandem mass tag (*TMT*) labeling of a sample pooled from the eight reactions, separation by liquid chromatography (*LC*), determining relative TMT tagging, adjustments of the sample volumes based on relative TMT representation, adjustment accordingly of volumes of the eight samples to generate a mixture of equal TMT representation, followed by a measurement by mass spectrometry (*MS*) and quantification of relative protein intensities by tags. Eight co-IP reactions were performed in parallel, with samples 1 and 2 being labeled with two different tags (Table 1) for normalization.

After TMT labeling of the immunoprecipitated samples, we performed an LC-MS/MS (liquid chromatography tandem MS) analysis (Fig. 2). This allowed us to identify Tau isoform-interacting proteins and to quantify these interactions. To ensure that the same amount of each labeled sample was injected into the mass spectrometer, the tag intensity was confirmed to be within a 20% deviation among the samples.

Our analysis first revealed that MS was able to detect the 0N, 1N, and 2N Tau isoforms (Table 2), in that, for example, the TMT ratio for Tau in the 1N IP reaction was 38% higher than for the KO IP reaction, indicating that the 1N antibody had successfully immunoprecipitated 1N Tau from the brain lysate. Interestingly, for MAP2 that belongs to the same MAP family as Tau, the TMT ratio was about four times higher in the Tau KO sample compared with the wild-type samples. This confirms previous findings that had shown an up-regulation of MAP1A in Tau KO mice (27), together demonstrating compensatory mechanisms when Tau is lacking.

**TABLE 2:** **TMT ratios for Tau and MAP2** CV means coefficient of variation.

Sample	Tau			MAP2		
	Denominator: IP:Dako with Tau KO lysate tagged with 127C			Denominator: IP:Dako with C57BL/6 lysate tagged with 126		
	TMT ratio	CV	Count	TMT ratio	CV	Count
IP:Dako/C57BL/6	5.65	58.9%	394	1.06	7.8%	40
IP:Dako with Tau KO	1	63.2%	394	4.22	37.4%	40
IP:Mouse/C57BL/6	5.81	56.4%	394	1.09	39.9%	40
IP:0N/C57BL/6	2.09	33.8%	394	0.82	23.6%	40
IP:1N/C57BL/6	1.38	40.4%	394	0.82	31.2%	40
IP:2N/C57BL/6	1.83	39%	394	0.88	31.2%	40

We next aimed to identify the proteins that interact with all three Tau isoforms. To achieve this, the protein quantities for the IP:Dako reactions of C57BL/6 and Tau-KO brain tissue were compared after normalization for the Dako anti-Tau antibody. Proteins for which the IP:Dako/C57BL/6 reaction gave readings that were at least 10% higher (and had a *p* value ≤0.05) than for the IP:Dako/Tau-KO reaction were considered to be Tau-interacting proteins. Thus, the list of 218 immunoprecipitated proteins was reduced to 101 Tau-interacting proteins (supplemental Table 1). It has to be noted that because of chemical background issues of the TMT method, the observed fold-change is always smaller than the actual fold-change (28). For example, a 10% change determined by TMT likely indicates a 20–50% actual change. As many of the changes in protein binding of the co-immunoprecipitated proteins were in the order of 20–50%, using a higher cutoff ratio may have eliminated many interacting proteins (supplemental Table 1).

To identify the binding preference of particular Tau isoforms, the MS data from the immunoprecipitated reactions using wild-type mouse brain lysates and the Dako, M, 0N, 1N, and 2N antibodies (IP:Dako, IP:M(ouse), IP:0N, IP:1N, and IP:2N) were normalized to the total intensity (*i.e.* amount) of the pulled down proteins. To normalize pulled down protein quantities, we generated a sample-specific protein database by exporting the identified proteins without antibodies. This identified the proteins that were co-immunoprecipitated with Tau, using the NCBInr *M. musculus* database. This pulled down protein database was used for TMT data processing and quantification. We employed this form of normalization because the antibodies differ in their affinities, and hence, no linear relationship can be established between the concentration of a particular antibody in the IP reaction and the amount of pulled down protein. The affinity of a particular antibody in an IP reaction is more appropriately reflected by its capacity to pull down bait-related proteins. Therefore, we excluded the reading from the antibodies used for IP and only used the total of the pulled down proteins for normalization. Unfortunately, the IP:normal serum data did not fit into the two normalization methods we had adopted, one of which uses the intensity of the Dako anti-Tau antibody as the denominator and the other the total pulldown with anti-Tau antibodies. Because the IP:normal serum reaction used normal mouse serum for the pulldown, which does not contain the denominator used above, we excluded the IP:normal serum data from the MS analysis and used the IP:Dako data from Tau KO mice as the sole negative control.

Our analysis revealed that 24 of the 101 identified proteins bind preferentially to 0N Tau (supplemental Table 2), 23 bind preferentially to 1N (supplemental Table 3), and 32 bind preferentially to 2N (supplemental Table 4), whereas 22 interact with all isoforms to a similar degree (supplemental Table 5). A protein was classified as preferentially binding when the binding ratio was at least 10% higher than for the other two isoforms. To name a few, we identified as 0N-interacting proteins ATP synthase β-subunit, α-synuclein, β-synuclein, mitochondrial creatine kinase U-type (MtCK), creatine kinase B-type, synapsin 1, and synaptogyrin-3; as 1N-interacting proteins ATPase, neuromodulin, tropomyosin α-1 chain isoform 10, calmodulin, and myelin basic protein isoform 3; as 2N-interacting protein apoA1, apoE, synaptotagmin, syntaxin 1B and 14-3-3ζ; and as proteins with no preference different ATPase subunits, cofilin-1, synaptophysin, and Dnm1 (also known as Drp1). However, we did not detect Fyn that is known to interact with Tau (see below).

##### Validations of Novel Interactions of Tau

**FIGURE 3.**
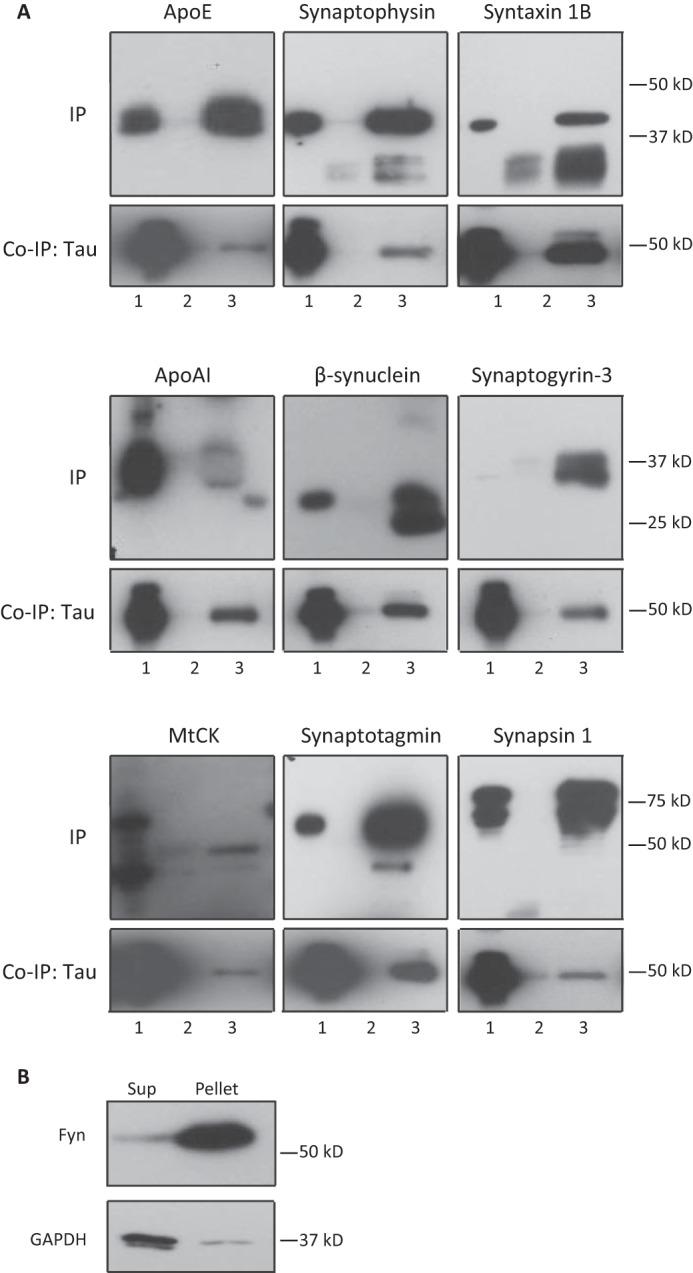
**Validation of identified Tau-interacting proteins by co-immunoprecipitation.** *A,* Tau-interacting proteins were immunoprecipitated under native conditions from brain lysate obtained from a 2-month-old wild-type mouse using antibodies for apoE, synaptophysin, syntaxin 1B, apoA1, β-synuclein, synaptogyrin-3, MtCK, synaptotagmin, and synapsin 1 and then blotted with an anti-Tau antibody. The input is shown in *lane 1*; the wild-type lysate that has been immunoprecipitated with normal IgG is shown as a negative control in *lane 2*; and the IP with the specified antibody in *lane 3. B,* Fyn that is known to interact with tau was not co-immunoprecipitated because of the buffer used for the reaction. Extraction of mouse brain tissue in IP buffer yielded a supernatant (*Sup,* input for IP) and a pellet that was further extracted with 70% formic acid to obtain the pellet fraction. 20 μg of supernatant fraction (the equivalent of that used in the above IP reactions) contains very little Fyn compared with the pellet.

To verify that the protein interactions identified by MS occur under native conditions, we performed co-IP reactions for several previously unreported Tau-interacting proteins. Our Western blot data revealed that antibodies against apoE, synaptophysin, syntaxin 1B, apoA1, β-synuclein, synaptogyrin-3, MtCK, synaptotagmin, and synapsin 1 all co-immunoprecipitated the respective proteins with Tau when wild-type mouse brain lysates were used (Fig. 3*A*). This confirmed that these protein interactions with Tau do indeed occur. We did not detect Fyn because of the buffer used for the IP reaction. Extraction of mouse brain in IP buffer yielded a supernatant (input for IP) and a pellet that was further extracted with 70% formic acid to obtain the pellet fraction. This revealed that the supernatant fraction (the equivalent of that used in our IP reactions) contained very little Fyn compared with the pellet (Fig. 3*B*).

##### Annotation and Classification of Tau-interacting Proteins

**FIGURE 4.**
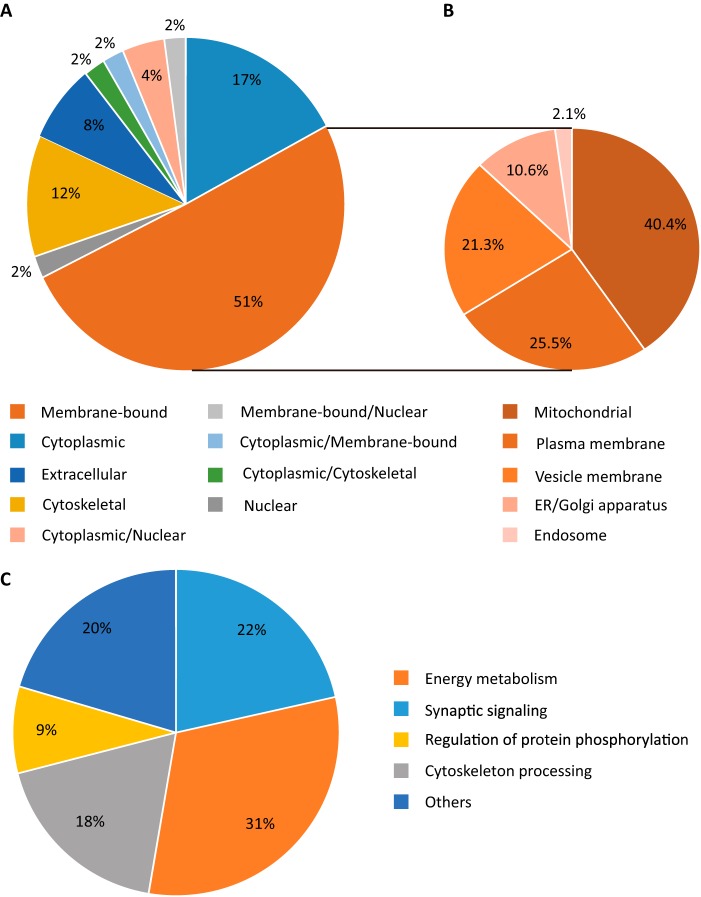
**Classification of Tau-interacting proteins.** Charts of the subcellular localization of the identified Tau-interacting proteins (*A*), the subset of membrane-bound proteins identified as the largest category using the annotation cell compartment (*B*), and functional classes of the identified proteins (*C*) are shown. Only major categories are shown; categories with fewer proteins are cumulatively displayed as *others*.

All proteins that had been identified in the IP reactions by MS were assigned to broad classifications based upon both known and predicted “subcellular locations, cell component, and function” using the UniprotKB protein knowledge database that was manually annotated and reviewed. Of the 101 proteins identified from the NCBInr database, 93 have reviewed annotations in UniprotKB (supplemental Table 6). In terms of the subcellular localization of Tau-interacting proteins, membrane-bound proteins composed the majority of identified interacting proteins (51%), followed by cytoplasmic (17%) and cytoskeletal proteins (12%) (Fig. 4*A*).

We next examined more closely the category of membrane-bound proteins with the term cell compartment. Interestingly, within the membrane-associated category, mitochondrial proteins composed the largest fraction (40.4%), followed by plasma membrane (25.5%), vesicle membrane proteins (21.3%), and proteins belonging to the endoplasmic reticulum/Golgi apparatus (10.6%) and the endosome (2.1%) (Fig. 4*B*). A separate functional classification of the proteins identified as major categories are energy metabolism (31%), followed by synaptic signaling (22%), cytoskeleton processing (18%), regulation of protein phosphorylation (9%), and others (20%) (Fig. 4*C*).

##### Protein-Protein Interaction Network for Tau and Tau-interacting Proteins

To access the degree of interaction of the identified Tau-interacting proteins, all listed proteins (supplemental Table 1) were imported into this database to establish a direct interaction network of the identified 101 proteins. One major Tau interaction cluster contained 33 Tau-interacting proteins that had been identified by MS (data not shown). Among the proteins were tubulin, actin, cofilin, dynamin, myelin basic protein, and heat shock protein 90 (Hsp90).

**FIGURE 5.**
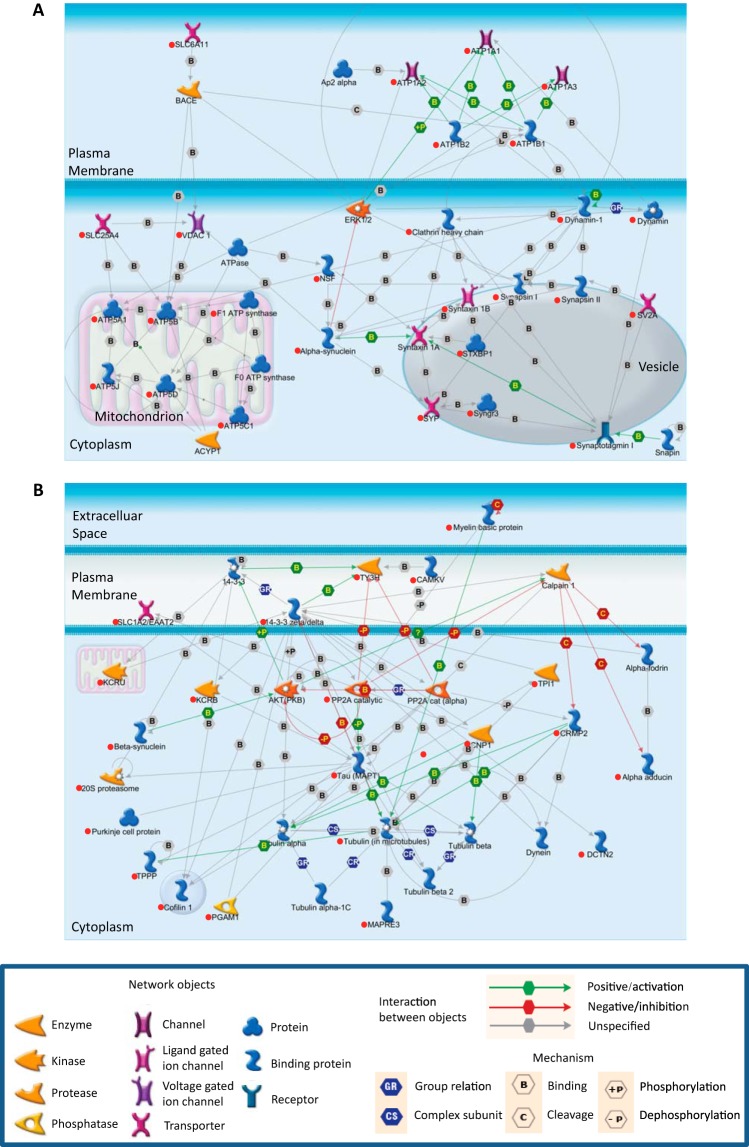
**Ingenuity pathway analysis identifies two highly enriched networks for the Tau-interacting proteins.** These networks are the cell-to-cell signaling and interaction (*A*) and the neurological disease network (*B*). Tau and its interacting proteins are marked with *red dots*.

**FIGURE 6.**
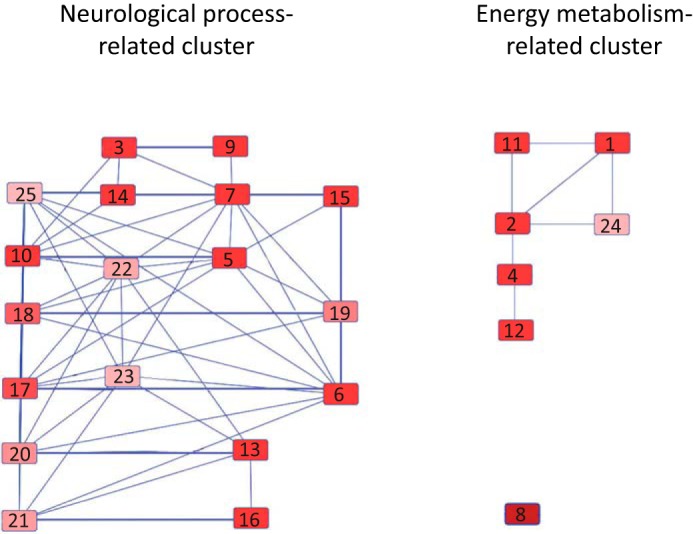
**Pathway cluster analysis.** The top 25 canonical pathways enriched for Tau-interacting proteins are as follows: *1*) glycolysis I; *2*) gluconeogenesis I; *3*) mitochondrial dysfunction; *4*) TCA cycle II (eukaryotic); *5*) remodeling of epithelial adherens junctions; *6*) clathrin-mediated endocytosis signaling; *7*) Huntington disease signaling; *8*) creatine-phosphate biosynthesis; *9*) oxidative phosphorylation; *10*) 14-3-3-mediated signaling; *11*) sucrose degradation (mammalian); *12*) isoleucine degradation I; *13*) glutamate receptor signaling; *14*) Parkinson disease signaling; *15*) GABA receptor signaling; *16*) glutamine biosynthesis I; *17*) gap junction signaling; *18*) germ cell-Sertoli cell junction signaling; *19*) virus entry via endocytic pathway; *20*) calcium signaling; *21*) amyotrophic lateral sclerosis signaling, *22*) breast cancer regulation by stathmin1; *23*) fMLP signaling in neutrophils; *24*) NADH repair; and *25*) axonal guidance signaling. The pathways were ranked by enrichment score (−log(P)) from high to low. The threshold of the enrichment score was 1.3.

To explore the pathways in which the interacting proteins in the major Tau-interacting cluster are involved, these proteins were subjected to an IPA. This revealed two significant overlapping pathways as follows: cell-to-cell signaling and interaction (Fisher's exact test, adjusted *p* = 1.14e-16) (Fig. 5*A*), and neurological disease (Fisher's exact test, adjusted *p* = 3.93e-18), which came out as enriched (Fig. 5*B*). The cell-to-cell signaling and interaction pathway highlights three groups of proteins as follows: (i) ATPase and ATP synthase that provide the energy for vesicular transport; (ii) proteins with a role in synaptic vesicle formation and docking, such as synapsin and synaptotagmin; and (iii) cytoskeletal proteins involved in vesicular transport, including actin and dynamin. The neurological disease pathway shows proteins that contribute to neurological disease. Of note are the Tau phosphatase PP2A and the Tau kinase AKT (PKB), among others. To reveal the relationship between pathways containing Tau-interacting proteins, all identified Tau-interacting proteins were loaded into IPA, and a map of relationships of the top 25 canonical pathways was generated using the Ingenuity knowledge base software. Two major pathway clusters were identified as follows: a neurological process-related cluster and an energy metabolism-related cluster. The link between the two pathways is determined by shared common components, for example, the 14-3-3 protein that has a role in both 14-3-3-mediated signaling (10) and axon guidance signaling (25); thus, 14-3-3 is a common component linking these two pathways (Fig. 6).

##### Proteins That Preferentially Bind to 2N Tau Are Specifically Associated with “Neurological Disease”

**FIGURE 7.**
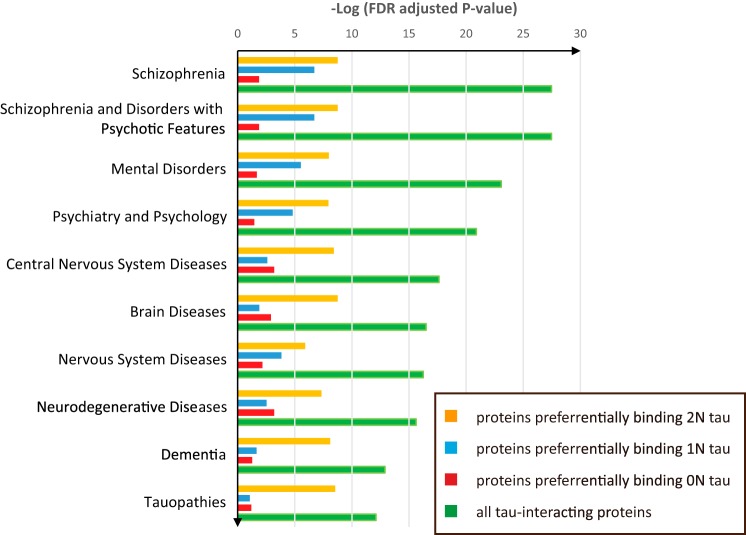
**Functional distribution and enrichment of Tau-interacting proteins classified according to “biomarkers of diseases” using Metacore analysis.**

Using the functional enrichment tool DAVID (29), we generated a list of enriched categories (in the three categories are biological process, cellular component, and KEGG pathway) for all Tau-interacting proteins identified by MS, both for proteins preferentially binding to specific Tau isoforms and for proteins interacting with all Tau isoforms with a similar binding ratio (Table 3). The enrichment categories of the biological process GO term for all identified Tau-interacting proteins included glycolysis, ATP biosynthetic process, synaptic transmission, cellular homeostasis, nucleotide binding, regulation of system process, and cellular respiration (Table 3). We also performed a functional enrichment of cellular component that yields the distribution of Tau-interacting proteins in cell compartments (Table 3). The KEGG pathway enrichment showed that Tau-interacting proteins were specifically associated with neurological diseases, such as Parkinson disease, Alzheimer disease, and Huntington disease (Table 3). Interestingly, KEGG enrichment analysis revealed that proteins preferentially binding 2N Tau were enriched in the neurological disease pathway (Table 3). To further confirm this observation, we used an enrichment for disease biomarkers that is run by MetaCore. Interestingly, for the top 10 enriched diseases, the enrichment score (−log (adjusted *p* value)) of the proteins that preferentially bind to 2N Tau was always the highest compared with those for the other two isoforms (Fig. 7). This suggests that although Tau-interacting proteins mainly participate in energy metabolism and neurophysiological processes, proteins that bind to 2N Tau had a more prominent role in neurological disease.

**TABLE 3:** **DAVID functional enrichment** A indicates enrichment of the biological process; B indicates the cellular component; and C indicates the KEGG pathway.

No.	Annotation cluster	Total	0N	1N	2N	Similar
**DAVID functional enrichment of biological process (Benjamini and Hochberg adjusted *p* value)**						
1	Glycolysis	3.76E-08	0.0047	0.0174		
2	ATP biosynthetic process	2.24E-08			0.0037	0.0092
3	Synaptic transmission	3.48E-07			0.0035	
4	Cellular homeostasis	3.64E-06	0.0043			
5	Nucleotide binding	0.0083				0.017
6	Regulation of system process	7.85E-05			0.0049	
7	Cellular respiration	0.0072	0.0480			

**DAVID functional enrichment of cellular component (Benjamini and Hochberg adjusted *p* value)**						
8	Mitochondrion	1.01E-05	0.0187		0.0073	
9	Vesicle	9.92E-04				0.0482
10	Synapse	0.0334	0.0222			
11	Cytoskeleton	0.0215				0.0490
12	Membrane	0.0197				0.0482

**DAVID functional enrichment of KEGG pathway (Benjamini and Hochberg adjusted *p* value)**						
13	Parkinson disease	0.0056			0.0073	
14	Alzheimer disease	0.0043			0.0121	
15	Huntington disease	0.0112			0.0082	
16	Glycolysis/ gluconeogenesis	1.38E-04	0.0488	0.0232		

##### Reverse-IP Validation of Selected Interactions

**FIGURE 8.**
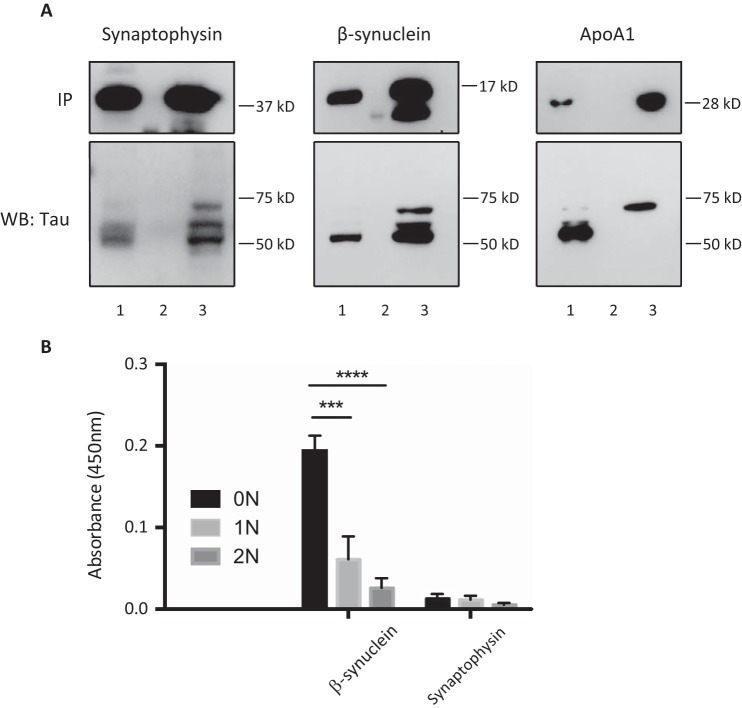
*A,* reverse-IP validation of selected interactions. To validate the binding preference of Tau-interacting proteins for specific Tau isoforms as identified by MS, dephosphorylated wild-type extracts (neither heated nor dialyzed) were immunoprecipitated with antibodies for synaptophysin, β-synuclein, and apoA1, respectively, followed by probing with the Dako Tau antibody. Quantification of the Western blot (*WB*) reveals a ratio of 0N:1N:2N = 1:0.97:0.93 for synaptophysin and 1:0.897:0.5 for β-synuclein. In the case of apoA1, only the 2N isoform was detected. There are three lanes per reaction: *lane 1,* input; *lane 2,* flow-through; and *lane 3,* IP. *B,* ELISA determination of direct interactions. To determine whether direct binding of Tau-interacting proteins to specific Tau isoforms occurs, ELISA was conducted using plates coated with recombinant 0N, 1N, and 2N Tau and then incubated with recombinant apoA1, β-synuclein, and synaptophysin. Binding was detected using either an anti-apoA1 antibody or an anti-V5 antibody for β-synuclein and synaptophysin. Measurements were conducted in triplicate (*n* = 2) and were analyzed after subtraction of the background absorbance. β-Synuclein binds preferentially to 0N Tau compared with 1N (***, *p* < 0.001) and 0N (****, *p* < 0.0001) Tau with a ratio of 0N:1N:2N = 1:063:026. Synaptophysin showed very weak binding to all isoforms of Tau, whereas apoA1 did not show any interaction with Tau.

To validate the binding preference of Tau-interacting proteins for specific Tau isoforms as identified by MS, we performed co-IPs for three Tau-interacting proteins, synaptophysin, β-synuclein, and apoA1, using dephosphorylated brain lysates (Fig. 8*A*). MS analysis had revealed a ratio of 0N:1N:2N = 1:1.1.07:1.09 for synaptophysin, suggesting no pronounced binding preference of synaptophysin for any of the three isoforms. When we quantified the Western blot of the reverse-IP, we obtained the ratio 0N:1N:2N = 1:0.97:0.93, confirming the MS analysis. For β-synuclein, MS had determined a ratio of 0N:1N:2N = 1:0.89:0.48, suggesting preferential binding for 0N Tau. The Western blot for the reverse-IP yielded a ratio of 0N:1N:2N = 1:0.41:0.37, again supporting the MS data. Finally, for apoA1, MS data gave the ratio 0N:1N:2N = 1:0.84:4.85, suggesting a very strong preference for 2N Tau in the interaction with apoA1. Remarkably, when we performed a Western blot analysis of the reverse-IP, we only detected the 2N isoform.

To validate some of the identified Tau interactions more directly, binding of recombinant 0N, 1N, and 2N Tau to recombinant apoA1, β-synuclein, and synaptophysin was determined using ELISA (Fig. 8*B*). Plates were coated with 0N, 1N, and 2N Tau, and bound apoA1, β-synuclein, or synaptophysin was detected using either an anti-apoA1 antibody or an anti-V5 antibody for β-synuclein and synaptophysin. Consistent with the MS data and reverse-IP (Fig. 8*A*), β-synuclein showed significant preferential binding to 0N Tau with a ratio of 0N:1N:2N = 1:063:0.26, demonstrating a direct interaction of β-synuclein with Tau. In contrast, synaptophysin showed very weak binding to all isoforms of Tau, and apoA1 did not bind at all. This suggests that additional co-factors may be required for the interaction between synaptophysin and apoA1 with Tau. To determine the functional relevance of these interactions, one is limited by the fact that it is difficult to predict whether any of the interactions of Tau would be affected by overexpression or removal of Tau, and whether this would show in differences in subcellular localization, expression levels, activity, and so forth, and also whether this would only show in particular brain regions and cell types.

Together, our findings highlight distinct protein interactions of the different Tau isoforms, suggesting that they execute different functions, which may be impaired to different degrees in neurodegenerative disease.

## Discussion

Using both novel Tau isoform-specific antibodies and commercially available pan-Tau antibodies, IP reactions followed by TMT labeling and quantitative MS identified 101 proteins that either directly or indirectly interact with Tau. We also identified preferential interactions for the three Tau isoforms, and importantly, we identified a distinct role for 2N Tau in neurological disease, using a set of database and pathway tools.

The Tau-interacting proteins that we have identified can be categorized into several groups according to their subcellular localization and biological function. Although Tau is well known for its interaction with microtubules, cytoskeletal proteins only accounted for one-tenth of all identified proteins and were ranked as the fourth largest category based on subcellular localization. In contrast, almost half of the Tau-interacting proteins were grouped as membrane-bound proteins, comprising the largest category. It is the projection domain of Tau (localized in its N-terminal half) that has a crucial role in the protein's membrane association (30). Its proline-rich domain contains P*XX*P motifs that facilitate the interaction with proteins containing Src homology 3 domains, such as Fyn (31, 32). Our data support accumulating evidence that Tau is associated with intracellular membranes, including the endoplasmic reticulum (33), the Golgi network (34), and the plasma membrane (35). However, we did not identify Fyn itself, because of the buffer used for the IP reaction. This points to a limitation inherent to all co-IP approaches in that a buffer needs to be used that (*a*) does not co-IP proteins that do not interact, but (*b*) does co-IP proteins that do interact, without disrupting their interaction (such as the Tau isoforms and their interacting proteins). Therefore, an IP is unlikely to identify the complete interactome of any protein of interest.

By assessing the subcellular compartments to which the membrane-bound Tau-interacting proteins localize, we found that plasma membrane proteins comprised the second-largest category, and mitochondrially localized proteins the largest. Mitochondrial localization is not surprising given that an impaired function of this organelle characterizes tauopathies such as AD (36, 37). In *Drosophila* and mouse models, it has been demonstrated that overexpression of Tau can induce cell death by elongating mitochondria; this is achieved by a reduced localization of the mitochondrial fission protein DRP1 to mitochondria (38), a protein identified in our screen. A study of the distribution of mitochondria in AD brains showed that their numbers were significantly decreased in Tau tangle-bearing neurons compared with neurons in healthy controls (39). Similar results were obtained in the Tau transgenic mouse strain rTg4510 (39). 3×Tg-AD mice, which exhibit both a Tau and an amyloid pathology, present with decreased mitochondrial respiration and decreased pyruvate dehydrogenase protein levels and activity (40). In ^triple^AD mice, both separate and synergistic effects of Tau and amyloid were found in relation to different mitochondrial functions (41). Our co-IP further demonstrated an interaction between Tau and the kinase MtCK. This enzyme is a central controller of cellular energy homeostasis, and its activity is highly susceptible to oxidative stress, with an 86% reduction observed in AD patients (42). Tau transgenic flies have an elevated superoxide production compared with wild-type controls, together with increased neuronal death (38). Combined with our data, this suggests that Tau may integrate oxidative stress signals, mitochondrial dysfunction, and neurodegeneration. It is possible that Tau may have an effect on mitochondrial functions not only by affecting reactive oxygen species levels but also by interacting with mitochondrial proteins such as MtCK.

When categorizing the Tau-interacting proteins according to their biological function based on annotation in UniProtKB, the top groups were synaptic signaling (31%), followed by energy metabolism, and cytoskeleton processing. In recent years, a synaptic role of Tau has entered the spotlight. Tau was found to target Fyn to the *N*-methyl-d-aspartic acid receptor, mediating amyloid-β-induced excitotoxicity at the synapse (31). Subsequently, a comprehensive study in three separate transgenic AD-related mouse models (hAPPJ9, hAPPJ20, and TASD41) showed that reducing Tau levels restores normal synaptic activity and network firing (43). More recently, a mis-sorting of Tau to dendritic spines was observed in P301L mice, leading to the loss of glutamate receptors and dampening of synaptic signaling (44). Although Fyn was not on the list of our identified Tau-interacting proteins, we identified another postsynaptic protein, scaffold protein 14-3-3ζ, that has been previously demonstrated to associate with Tau and function as an effector of Tau phosphorylation (45). More recently, several studies have shown that the 14-3-3 isoforms γ, θ, and ζ provide a platform for glycogen synthase kinase-3 (GSK-3) in regulating Tau phosphorylation, participating in protein trafficking, and also facilitating signal transduction of exocytotic pathways (46, 47). In addition, 14-3-3 was shown to bind the postsynaptic scaffolding protein PSD-95 that is highly expressed in hippocampal glutamatergic synapses involved in the Fyn-Tau interaction, establishing an indirect interaction between 14-3-3 and glutamate receptors, K^+^ channels, and scaffolding and signaling proteins (48). Similarly, 14-3-3 was found to interact with glutamate receptors via binding to homer homolog 3 (Homer 3). It also plays a role in cytoskeletal reorganization and spine morphogenesis as it can induce the maturation of spines in cultured rat hippocampal neurons (49). Together with our findings, this suggests that 14-3-3 may not only function as an effector of Tau phosphorylation but also provide a platform for Tau to interact with other signaling proteins.

It is worth noting that in addition to the postsynaptic scaffolding protein 14-3-3, many presynaptic vesicle-related proteins were identified by our MS analysis, such as synapsin I, synaptophysin, synaptogyrin-3, synaptotagmin, and syntaxin-1B. These proteins are all involved in neurotransmission and are critical for vesicle docking and fusion. Although a direct interaction between Tau and synaptic vesicle proteins still needs to be demonstrated, these five presynaptic proteins were immunoprecipitated by us in five separate reactions using different anti-Tau antibodies and subsequently verified by co-IPs. This is robust evidence for a role of Tau in presynaptic signal transduction. Synapsin I is an actin-bundling protein that regulates the clustering of synaptic vesicles and exocytosis together with actin (50). Tau has also been shown to interact with actin *in vitro* and may mediate neuronal degeneration by altering the organization and dynamics of the actin cytoskeleton (51). Together with our findings, this presents the possibility that Tau interacts with synapsin I via actin and thereby also alters actin dynamics and presynaptic vehicle transport, which may result in synaptic failure and neurodegeneration. In addition, Tau may interact with another synaptic vehicle protein, syntaxin 1B, via dynamin-1. An interaction of Tau with these two proteins has been reported in secretory granules (52). Syntaxins 1A and 1B are co-expressed in neurons, and a recent study proved that syntaxin 1B, but not syntaxin 1A, is crucial for the regulation of synaptic vesicle exocytosis (53). Hoover *et al.* (44) mutated 14 phosphorylation sites in a proline-rich region of Tau to negatively charged glutamate residues to mimic phosphorylation; this resulted in a more frequent accumulation of the mutated Tau in dendritic spines than wild-type Tau and disrupted synaptic functions. Moreover, double transgenic mice expressing the P301S Tau mutation together with a mutation found in familial Danish dementia showed a significant increase of Tau deposition and a significant decrease in synaptophysin levels in mouse brain (54). Although Tau has not been shown to directly interact with these synaptically related proteins, the above earlier studies showed that it may interact with synapsin I, syntaxin 1B, and other synaptic proteins (or a complex formed by these synaptic proteins) via cytoskeletal proteins and impact on synaptic transmission through these indirect interactions. We have recently shown that Tau is localized to the spine head of hippocampal neurons and that this localization is facilitated by pseudo-phosphorylation of Tau at distinct sites (55). Further studies are required to determine whether Tau interacts directly with syntaxin 1B and other synaptic proteins (with ELISA showing such an interaction for synaptophysin, see below).

Our co-IP also showed an interaction between Tau and apoE as well as apoA1 (with Tau isoform-specific interactions discussed further below). The apoE4 allele is a major risk factor for AD (56), with apoE being detected in neurofibrillary tangles (57). Interestingly, apoE3 and Tau have been shown to interact even under the presence of 2% SDS (58), and our finding that apoE is a Tau-interaction protein is consistent with this observation. More recently, apoA1 has been identified as marker of AD, with the concentration of apoA1 in cerebrospinal fluid distinguishing AD from non-demented subjects with 84% sensitivity and 72% specificity, with 78% of the subjects being correctly classified (59). By comparison, using Aβ_42_ alone gave 79% sensitivity and 61% specificity, with 68% of the subjects being correctly classified. Apolipoproteins regulate lipids, including cholesterol. Which role Tau has in lipid metabolism is not fully understood; however, our identification of apoA1 as a Tau-interacting protein makes these links stronger.

Two additional interesting Tau-interacting proteins identified by MS are α- and β-synuclein, the former forming aggregates in Lewy bodies, a hallmark lesion of Parkinson disease. The two synucleins show a similar expression level in the brain and co-localize in presynaptic terminals (60, 61). Earlier affinity chromatography experiments had shown direct binding between α-synuclein and Tau (62). Our co-IP study followed by an ELISA further revealed a direct interaction between β-synuclein and Tau. It has been hypothesized that Tau and α-synuclein promote each other's accumulation, a sign of co-morbidity (63). A growing number of studies support this hypothesis, with Tau shown to enhance the aggregation of α-synuclein (64).

Whereas direct protein/protein interactions are typically determined by techniques such as fluorescence resonance energy transfer, surface plasmon resonance, or pulldown assays with purified proteins, MetaCore, which uses a proprietary database to reveal direct protein/protein interactions, identified a cluster of proteins centered on Tau, in which three proteins, tubulin, heat shock protein 90 (Hsp90), and calcineurin, also known as PPP3, are shown to bind Tau directly. The map also shows that tubulin not only directly binds Tau but also binds actin and dynamin-1, which is associated with synapsin I and synapsin II and provides a bridge between Tau and synaptic vesicle proteins. This map is consistent with our analysis of Uniprot annotations and our co-IP results. Moreover, Hsp90 is a major cellular chaperone that assembles large chaperone complexes. Hsp90 complexes function to maintain protein quality control and assist in protein degradation. When Tau becomes hyperphosphorylated and forms aggregates, a complex is formed between Hsp90 and Tau that triggers the ubiquitination of Tau and activates its degradation (65, 66). To further dissect the indirect interactions of those proteins that closely interact with Tau, a canonical pathways analysis was performed using the IPA database. This identified two highly enriched pathways, cell-to-cell signaling and interaction and neurological disease. The former encompasses a group of proteins involved in synaptic vesicle formation, docking, and fusion, with an important role in neurotransmitter release, whereas the second group includes proteins related to changes in neurological disease. Some of these are highly correlated with disease progress, such as Tau. The fact that Tau-interacting proteins are highly enriched in these two pathways would indicate that Tau is a critical component of them.

A novel finding of our study is also the identification of proteins with preferential binding to distinct isoforms of Tau, assisted by the use of isoform-specific antibodies and validated for a subset of these proteins by reverse IPs. By grouping the identified proteins based on their binding preference, we obtained four groups as follows: one each with preferred binding to either of the three Tau isoforms and one with no preference. By using the DAVID functional enrichment tool, different enrichment levels were calculated for these groups. This showed that the proteins that preferentially bind to 0N Tau are enriched in glycolysis, cellular homeostasis, and the cellular respiration pathway; proteins that preferentially bind to 1N Tau are enriched in glycolysis; and proteins that preferentially bind to 2N Tau are involved in the ATP biosynthetic process, synaptic transmission, and the regulation of the system process pathway. This finding is consistent with our previously published isoform localization study (11).

Among the validated Tau-interacting proteins are synaptophysin, β-synuclein, and apoA1. This revealed that the ratio obtained by MS analysis is reflected by that obtained by Western blotting of the reverse IP reaction. For synaptophysin, MS revealed a ratio of 0N:1N:2N = 1:1.1.07:1.09 and the reverse IP one of 1:0.97:0.93, suggesting no pronounced binding preference of synaptophysin for any of the three isoforms. For β-synuclein, MS had determined a ratio of 1:0.89:0.48 and a reverse IP of 1:0.41:0.37, suggesting preferential binding for 0N Tau. Finally, for apoA1, the MS data had given a ratio of 1:0.84:4.85, whereas by reverse IP, only 2N was detected, suggesting a very strong preference for 2N Tau in the interaction with apoA1.

Interestingly, the DAVID functional enrichment of the KEGG pathway demonstrated that proteins that preferentially bind 2N Tau are more enriched in Parkinson disease (adjusted *p* = 0.0073), Alzheimer disease (adjusted *p* = 0.0121), and Huntington disease (adjusted *p* = 0.0082), whereas proteins that preferentially bind 0N and 1N Tau showed no significant enrichment. However, proteins that preferentially bind 0N and 1N Tau are enriched in glycolysis and gluconeogenesis, which is consistent with the enrichment result using the GO term of biological process. To verify the finding that the proteins that preferentially bind 2N Tau are more enriched in these neurodegenerative diseases, a second functional enrichment analysis was conducted with MetaCore using disease biomarkers as the identifier, which again yielded consistent results. For neurodegenerative disease, dementia, and tauopathies, the proteins that preferentially bind 2N Tau all had higher enrichment scores. Thus, for the first time, proteins associated with a particular Tau isoform have been shown to be highly enriched in a disease-related pathway.

## Author Contributions

C. L. and J. G. conceived and coordinated the study; C. L., X. S., and R. N. performed the experiments. All authors analyzed and interpreted the data. C. L. and J. G. wrote the manuscript with contributions from X. S. and R. N.

## Supplementary Material

Supplemental Data

## Footnotes

2 The abbreviations used are:

MAP: microtubule-associated protein
AD: Alzheimer disease
apoA1: apolipoprotein A1
apoE: apolipoprotein E
GO: gene ontology
IP: immunoprecipitation
IPA: ingenuity pathway analysis
PP2A: protein phosphatase 2A
TMT: tandem mass tag.
