# Supplementary material for: Co-immunoprecipitation with Tau Isoform-specific Antibodies Reveals Distinct Protein Interactions and Highlights a Putative Role for 2N Tau in Disease
Source: J Biol Chem. 2016 Feb 9;291(15):8173–88. doi: 10.1074/jbc.M115.641902 (PMC4825019; doi:10.1074/jbc.M115.641902)
Supplement: Supplemental Data [file supp_291_15_8173__index.html]

Co-immunoprecipitation with tau isoform-specific antibodies reveals distinct protein interactions, and highlights a putative role for 2N tau in disease — Co-immunoprecipitation with Tau Isoform-specific Antibodies Reveals Distinct Protein Interactions and Highlights a Putative Role for 2N Tau in Disease — Tau Isoform-specific Protein Interactions — Supplemental Data 

# Co-immunoprecipitation with Tau Isoform-specific Antibodies Reveals Distinct Protein Interactions and Highlights a Putative Role for 2N Tau in Disease

## Supplemental Data

- Suppl. Table 1 (.xlsx, 24 KB) - Suppl. Table 1
- Suppl. Table 2 (.xlsx, 10 KB) - Suppl. Table 2
- Suppl. Table 3 (.xlsx, 10 KB) - Suppl. Table 3
- Suppl. Table 4 (.xlsx, 10 KB) - Suppl. Table 4
- Suppl. Table 5 (.xlsx, 10 KB) - Suppl. Table 5
- Suppl. Table 6 (.xlsx, 14 KB) - Suppl. Table 6
